# Microbiota Comparison of Amur ide (*Leuciscus waleckii*) Intestine and Waters at Alkaline Water and Freshwater as the Living Environment

**DOI:** 10.3389/fmicb.2022.881132

**Published:** 2022-05-04

**Authors:** Liang Luo, Yue Xu, Yumei Chang, Bo Sun, Limin Zhang, Zhigang Zhao, Liqun Liang

**Affiliations:** ^1^Key Laboratory of Cold Water Fish Germplasm Resources and Multiplication and Cultivation of Heilongjiang Province, Heilongjiang River Fisheries Research Institute of Chinese Academy of Fishery Sciences, Harbin, China; ^2^The Centre of Marine Sciences, University of Algarve, Faro, Portugal

**Keywords:** *Leuciscus waleckii*, intestine, microbiota, alkaline water, freshwater

## Abstract

The intestinal microbiota of marine animals was influenced by the water and environment in which they live. The Amur ide (*Leuciscus waleckii*) adapts to extremely high alkalinity and is an ideal material for aquacultural studies of alkaline adaptation. In this study, we screened intestinal indicator flora and functional redundancy of intestinal colonies in alkaline-water species (AW) and freshwater species (FW) of Amur ide (*L. waleckii*) in these different aquatic environments. The available vs. community composition correlations were then predicted by contrasting each other with the flora contained in environmental water samples. Here, five microbial species and six genera were identified owing to the classifiable sequence. The intestinal microbiota that existed in AW and FW had approximately 1/3 of the operational taxonomic units in the respective living water environments, meaning gut microbes in the aqueous habitats will have an influential association with gut microbes in AW and FW. Compared to the bacterial composition of the FW intestine and that present in freshwater, *Moraxella osloensis, Psychrobacter maritimus*, and *Psychrobacter faecalis* were significantly enriched in the intestine of AW and alkaline water samples. In the FW intestine and freshwater samples, however, *Cryptomonas curvata* and *Polynucleobacter asymbioticus* were highly improved, which can be summarized as *Enterobacter sp*., the predominant population in the AW gut, while *Aeromonas* and *Ralstonia* being primarily present in FW intestines. Photosynthetic bacteria were most significant in both water samples. The results indicated that the intestinal microbiota composition, abundance, and diversity of AW and FW were quite different. In contrast, the microbial composition of the additional alkaline water and freshwater environments showed slight differences. This study expects to enhance our understanding of the alkalinity tolerance of *L. waleckii*, which will be provided for the breeding of fish living in alkaline water, and push the development of alkaline water resources with increased efficiency.

## Introduction

In contrast to terrestrial animals, fish must exhibit efficient ion and osmolality conditioning to comply with the unconventional changes in salinity, alkalinity, and ion composition of alkaline water and freshwater (Hwang and Lee, 2007). Multiple studies have shown that *Leuciscus waleckii* living in Dali Nor-Lake, Inner Mongolia, China, also known as alkaline water species (AW), can tolerate harsh water conditions with high alkalinity of ~53.57 mmol/L (pH 9.6) (Chang et al., 2013). On the other hand, freshwater species of *L. waleckii* (FW) living in freshwater at approximately pH 7.44, such as the Songhuajiang River and Heilongjiang River, became an ideal counterpart of AW due to geographical isolation and environmental elements for year-round impaction (Xu et al., 2013; Chang et al., 2014; Chen et al., 2019), raising broad concerns.

*L. waleckii* can rapidly adapt to salinity, and alkalinity changes in different water environments and has a high tolerance threshold for adaptation to alkaline water environments (Wang et al., 2021; Luo et al., 2022). It can interconvert from freshwater to alkaline water *via* a comparatively postponed directional domestication. The intestine is an essential organ for conditioning fish osmotic pressure during this process. It can efficiently exchange fluids to compensate for dehydration caused by hyperosmotic environments (Takei, 2021), which host various microorganisms having complex community structures. During long-term evolution, gut microbes have formed interdependent and mutually constrained relationships with their host's chronotype, influencing the host's susceptibility to infection by exogenous pathogens (Abid et al., 2013; Cahenzli et al., 2013). Thus, the gut microbiota serves as a virtual endocrine organ that defends against pathogen invasion, becoming a route for host nutrient acquisition to provide supplementation (Fan et al., 2019). However, saline-alkaline as a standard parameter affects physiological stress in fish, and changes in thermal pressure and osmolarity occur when saline-alkaline exceeds the tolerance range (Liao et al., 2015). The normal physiological function is altered and triggers subsequent energy-consuming stress responses and multiorgan dysfunction (Polinski et al., 2021) where endocrine pathways are present, leading to multiple stress-induced diseases or death in fish. Recently, it has been shown that saline-alkaline aquatic environments can affect intestinal bacteria (Zhang et al., 2016). However, the effect of alkaline changes paired with the water environment on the intestinal microbiota is unknown.

Therefore, this study aimed to compare the microbiota of the *L. waleckii* gut and water samples under alkaline water and freshwater *via* high-throughput sequencing and attempted to reveal some functional predictions using Phylogenetic Investigation of Communities by Reconstruction of Unobserved States (PICRUSt). Our main goals were as follows: (a) differences in the gut microbial composition, abundance, and diversity of AW and FW compared with their situation in alkaline water and freshwater environments, respectively; (b) exploring which primary gut microbial composition can change the function of AW and FW; and (c) to determine the relationship between microbiota composition in the intestines of AW, FW, and water samples. The results of this study will help reveal the role of the intestinal flora of alkaline water and freshwater species of *L. waleckii* from the simple structural analysis of intestinal flora to the exploration of their physiological function and provide a scientific basis for the development of artificial diets and probiotics in aquaculture.

## Materials and Methods

### Collection of Samples

**Figure 1:**
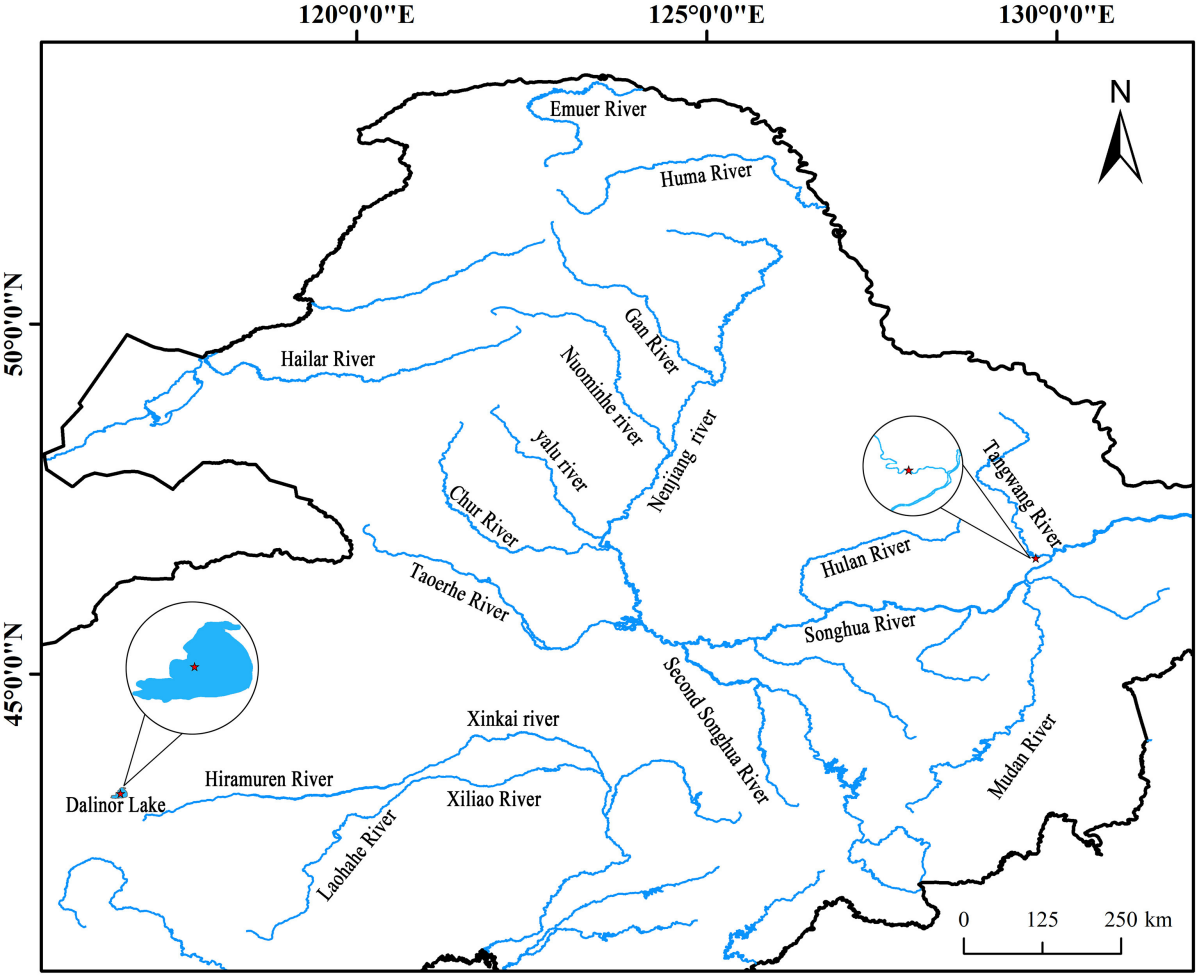
Geographical distribution of the sampled populations. Cycles represent the sampling locations and include alkaline water species (AW) from the Dali Nor lake and freshwater species (FW) from the Tangwang River.

Intestinal AW of *L. waleckii* and alkaline water samples were collected from Dali Nor-Lake (116°40′13″ E, 43°24′37″ N), Inner Mongolia, China (Figure 1), which were designated as DLG 1-3 and DLS 1-3, respectively. The living alkaline water conditions were water temperature of 24°C−25°C, pH of 9.4–9.6, the dissolved oxygen level of 8.86–9.34 mg/L, and alkalinity of 52.8–53.6 mmol/L. In addition, intestinal FW of *L. waleckii* and freshwater samples labeled as TRG 1-3 and TRS 1-3 respectfully were taken from the Tangwang River (129°44′24″ E, 46°41′15″ N) belonging to the Songhuajiang River tributaries, Heilongjiang Province, China (Figure 1). The living freshwater conditions were water temperature of 23°C−25°C, pH of 7.3–7.9, the dissolved oxygen level of 7.56–10.25 mg/L, and alkalinity of 0.5–0.8 mmol/L. Due to the influence of feeding habits and water temperature of *L. waleckii*, all the samples were collected in August when the bait was most abundant.

The water samples were collected using an aquatic microbial collection method (Li et al., 2017). Microbiological specimens were collected at 0.22 μm filter membrane *via* vacuum filtration, and the filter membrane containing the whole water sample was maintained in 15 ml sterilized centrifuge tubes, froze in liquid nitrogen, and then stored at −80°C for use. Sixty healthy adult fish (average 398.56 ± 13.58 g) taken from both locations above were quickly brought back to the sterile laboratory after being anesthetized and placed on a sterile operating table. The fishes' surface was sterilized with 75% ethanol, and the intestine was aseptically dissected. Each intestine sample is mixed with 10 healthy fishes intestines. The intestine was put into a 1.5 ml sterile centrifuge tube, froze in liquid nitrogen, and then stored at −80°C for use (Fan et al., 2019).

### DNA Extraction

The pure Soil DNA Mini Kit (Magen, Guangzhou, China) was used for microbial DNA extraction as per the instructions of the manufacturer. The proposed DNA concentration and purity were determined using a NanoDrop 8000 spectrophotometer (NanoDrop Technologies, Wilmington, USA). The conditions for PCR amplification of the V3–V4 region of the 16S rRNA were as follows: 94°C for 2 min, 98°C for 10 s, 62°C for 30 s, 68°C for 30 s for 30 cycles, and 68°C for 5 min. The primers used were 341 F (CCTACGGGNGGCWGCAG) and 806 R (GGACTACHVGGGTATCTAAT) (Guo et al., 2017), which were subjected to three PCR replicates as counterparts. The PCR amplification system followed by ReverTra Ace-α-™ (TOYOBO, Japan) contained the following: 5 μl 10 × KOD buffer, 5 μl 2 mM dNTPs, 3 μl 25 mM MgSO_4_, forward and reverse primers in 1.5 μl each (10 μM), 1 μl KOD polymerase, 100 ng DNA template, and appropriate volume of ddH_2_O obtaining a total reaction volume of 50 μl. All the PCR products were detected using 2% agarose gels containing ethidium bromide and were purified with the AxyPrep DNA Gel Extraction Kit (Axygen Biosciences, Union City, CA, USA), and quantified using QuantiFluor™-ST (Promega, USA). Purified PCR amplicons were pooled in equimolar and paired-end sequenced (PE250) using an Illumina MiSeq platform (Illumina, San Diego, CA, USA) according to the instructions of the manufacturer. The raw sequencing reads of all samples were deposited to the NCBI database under BioProject accession number: PRJNA810745.

### Library Preparation and Sequencing

Sequencing libraries were generated using the TruSeq® DNA PCR-Free Sample Preparation Kit (Illumina, USA) following the recommendations of the manufacturer, and index codes were added. Next, the library quality was assessed on a Qubit@ 2.0 Fluorometer (Thermo Scientific) and Agilent Bioanalyzer 2100 system. Finally, the library was sequenced on an Illumina NovaSeq platform, and 250 bp paired-end reads were generated.

### Data Analysis

The process comprises five main steps: data split, sequence assembly, filtration, chimera removal, and gene function prediction. Briefly, raw fastq sequence files produced in the 18.0 version were demultiplexed, quality-filtered by Trimmomatic, and concatenated by FLASH (Mago and Salzberg, 2011). Cluster sequences with similarities higher than 97% were assigned to the same operational taxonomic units (OTUs) (Caporaso et al., 2010). The most abundant sequence in each OTU was aligned with the Greengene database (DeSantis et al., 2006) to obtain taxonomic information on the OTUs. Using UPARSE (version 7.1 http://drive5.com/uparse/), OTUs were grouped with a 97% similarity criterion, and chimeric sequences were identified and deleted using UCHIME. Based on the Silva16S rRNA database (SSU123), the taxonomy of each 16S rRNA gene sequence was assessed by the RDP Classifier algorithm (http://rdp.cme.msu.edu/) against the Silva 16S rRNA database (SSU115) with a confidence level of 70% (Quast et al., 2012; Amato et al., 2013).

### Prediction of Gene Function

Phylogenetic Investigation of Communities by Reconstruction of Unobserved States PICRUSt (http://picrust.github.io/picrust/) is a biocomponent analysis package that aligns microbial community richness and databases by predicting microbial functions and metabolic pathways based on 16S rRNA high-throughput sequencing results. The useful Kyoto Encyclopedia of Genes and Genomes community information *via* the corresponding OTU table after aligning the sequencing data through the Greengenes database 13.8, and then homogenizing each out abundance (Loudon et al., 2014).

### Statistical Analysis

Based on the characteristics of the amplified regions, small fragment libraries were built for single-end sequencing utilizing the single-end sequencing method based on the IonS5TMXL sequencing platform, according to the principle of 16S rDNA amplicon sequencing technology. Furthermore, the *t*-test method for independent samples was used to compare differences in microbial diversity and relative abundance of bacteria in the analyzed intestines of AW, FW, and water samples. Principal coordinates analysis and analysis of similarity based on distances of unweighted UniFrac, Jaccard, and Bray–Curtis were performed using the R language vegan package (Conway et al., 2017) to compare their groups of samples in terms of differences and significance detection, respectively, in the composition structure of microbial communities.

## Results

### Statistical Analysis of Sequences

**Figure 2:**
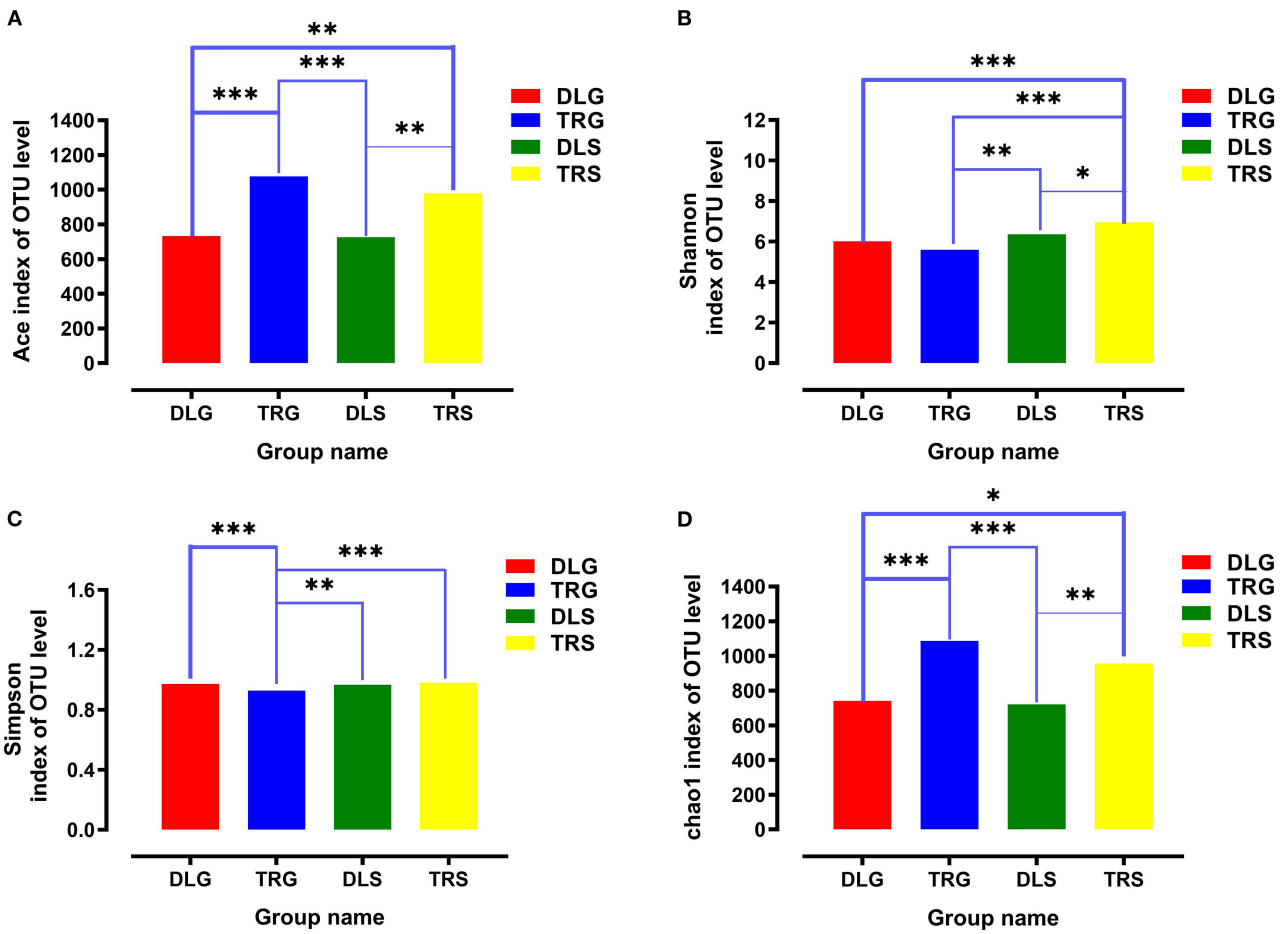
The richness and diversities of bacterial species in the four groups. **(A–D)** These figures are the Ace, Shannon, Simpson, and Chao index of OTU level separately. * stands for 0.01 < *P* ≤ 0.05, **stands for 0.001 < *P* ≤ 0.01 and ***stands for *P* ≤ 0.001.

For all samples, 16S rRNA gene V3–V4 regions of bacteria were sequenced to profile the microbiota of the intestines of AW and FW in alkaline water and freshwater, respectively, using the Illumina MiSeq platform. The initial data quality and chimeric filtering delivered 883,017 high-quality sequencing reads from 12 samples split into four groups (three samples per group) followed by 10 fishes per sample (Table 1 and Supplementary Table S1). In Figure 2, the richness and diversity of bacterial species are split into four groups: ACE, Chao, Shannon, and Simpson indices related to OTU level. The Shannon and Simpson indices are commonly used to quantify biodiversity. The richness of each sample was determined using the Chao index and ACE indices (Fan et al., 2019). The estimators of ACE ranged from 677.729 to 1224.136, Chao ranged from 727.238 to 1226.519, Shannon ranged from 5.390 to 7.079, and Simpson ranged from 0.930 to 0.989 (Table 1). These were summarized with a significant difference (*P* < 0.05) from each sample at a genetic distance of 3%. The results revealed that the microbial richness and diversity were significantly reduced in the alkaline water group compared to the freshwater group. The Good's coverage within each sample to calculate sequence thoroughness was an average of 99.3325%, indicating that the sequences discovered could represent the majority of microorganisms in each group. The data for the *t*-test can be found in Appendix S1.

**Table 1:** Richness and diversity indexes relative to each sample (OUT cutoff of 0.03)^a^.

**Sample ID**	**Read numbers**	**Coverage**	**Number of OUTs**	**Alpha diversity**			
				**ACE**	**Chao**	**Shannon**	**Simpson**
DLG1^b^	51898	0.998	682	719.832	727.238	6.449	0.971
DLG2	80316	0.996	666	791.44	793.991	5.718	0.989
DLG3	82980	0.997	594	682.288	701.639	5.888	0.963
TRG1	80182	0.995	851	1,050.436	1,067.496	5.546	0.946
TRG2	54944	0.995	1,045	1,224.136	1,226.519	5.844	0.93
TRG3	80184	0.996	856	956.533	963.79	5.39	0.909
DLS1	80149	0.997	609	732.673	740.211	6.417	0.969
DLS2	56961	0.998	599	677.729	660.348	6.345	0.968
DLS3	80400	0.997	608	770.233	765.08	6.325	0.967
TRS1	80222	0.996	878	988.458	967.877	7.024	0.982
TRS2	80185	0.995	889	1,065.644	1,031.245	7.079	0.984
TRS3	74596	0.996	755	883.959	870.874	6.761	0.977

a *OTUs were defined at the 97% similarity level (threshold is 0.03)*.

b *DL stands for Dali Nor-Lake, TR stands for Tangwang River, and G stands for Amur ide intestines, S stands for water*.

### Structure of Bacterial Community

**Figure 3:**
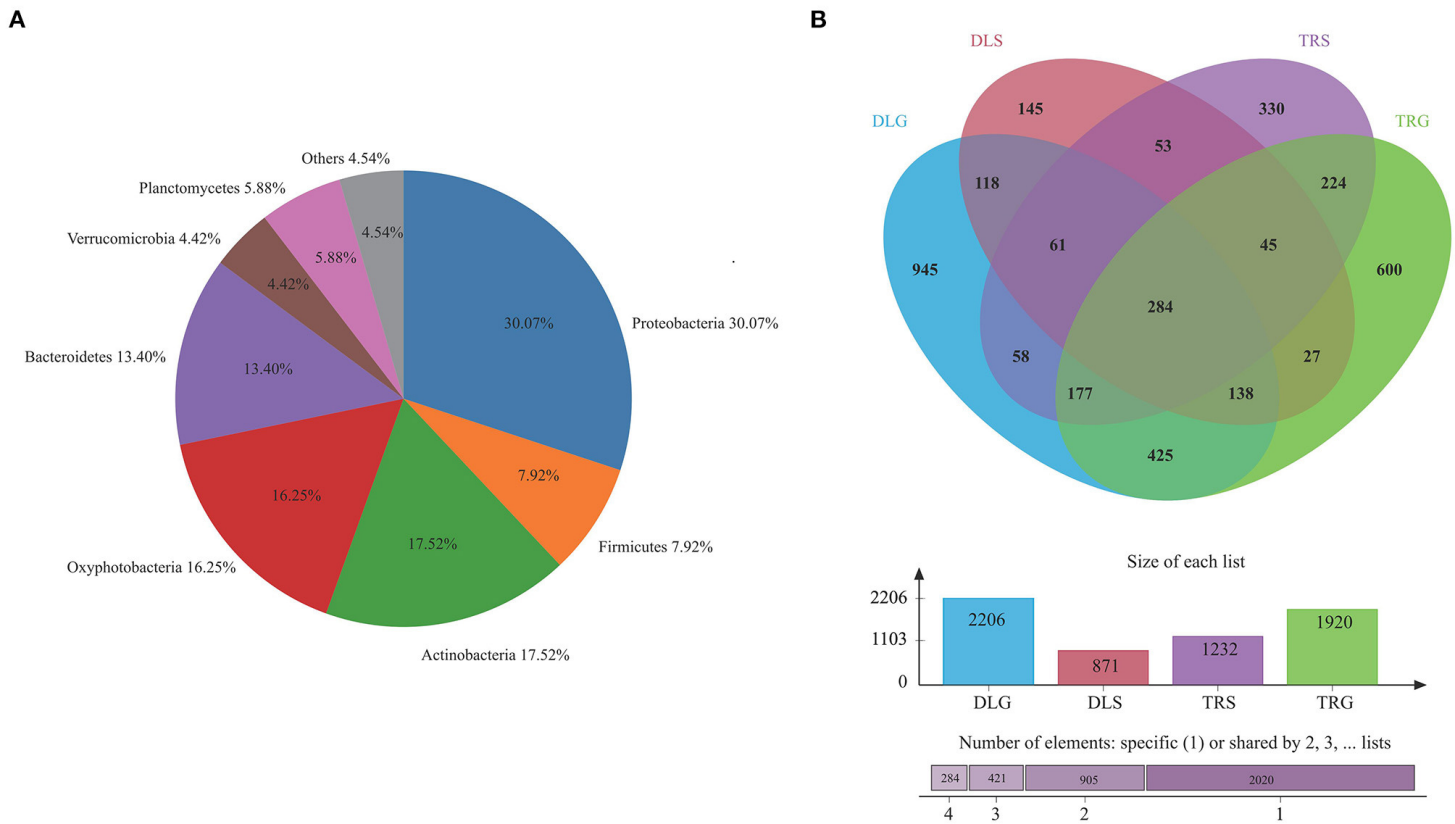
The bacterial community in all samples at the phylum level **(A)** and comparison of OTUs in the four groups by Venn diagram **(B)**. Others mean the sum of phylum or genus that relative.

The major phylum and genus relative abundance in intestinal bacteria of all samples from alkaline water and freshwater groups are summarized in Figure 3A, and any known sequences' groups are labeled as “others.” All the samples followed abundant phylum in descending order: *Proteobacteria* (30.07%) > *Actinobacteria* (17.52%) > *Oxyphotobacteria* (16.25%) > *Bacteroidetes* (13.40%) > *Planctomycetes* (5.88%) > others (4.54%) > *Verrucomicrobia* (4.42%).

Operational taxonomic unit (OTU) abundance was normalized using a standard sequence number corresponding to the sample with the most miniature sequences. Next, alpha and beta diversity analyses were performed based on the normalized output data. The Venn diagram in Figure 3B was developed to identify prominent OTUs provided in the four groups above to explore the significant microbiome in all samples between intestines and water types in alkaline water and freshwater environments. There were 284 OTUs shared among DLG, DLS, TRG, and TRS (*n* = 3), representing 4.64% of the total reads; sequencing by the number of OTUs was TRS > DLG > TRG > DLS, and the high-quality sequences were grouped into 1,467 OTUs at 97% similarity level (Figure 3B). Among them, 601 OTUs in the alkaline water group (DLG and DLS) were identified based on a similarity of <730 OTUs inside the freshwater group. Whereas 1,024 OTUs in intestinal samples of *L. waleckii* (DLG and TRG) were defined totally (specialized OTUs were, respectively, 1,182 and 896), based on a similarity more remarkable than the total 443 OTUs in alkaline water and freshwater (DLS and TRS) had specialized OTUs were, respectively, 428 and 789.

**Figure 4:**
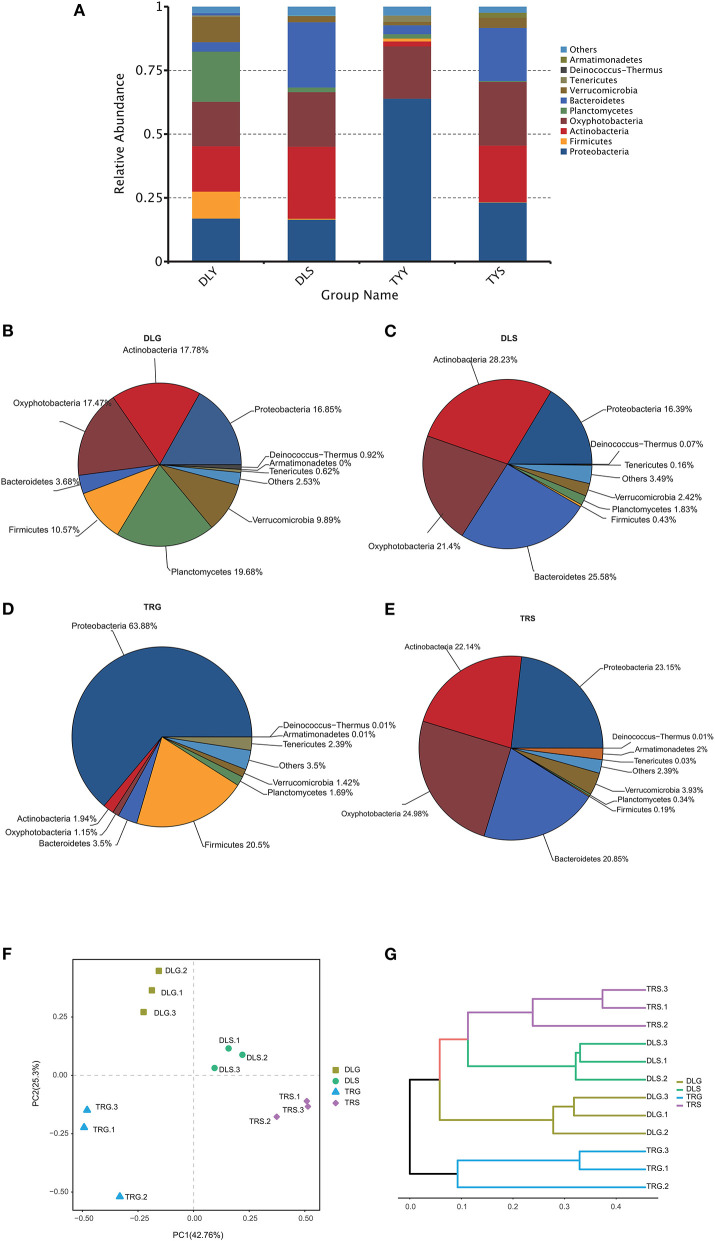
The microbiota composition at the phylum level **(A–E)**, the principal coordinates analysis (PCoA) of the bacterial community **(F)** at OTU level, and hierarchical clustering tree **(G)** in the four groups. Microbiota composition of bacterial taxa at phylum level in the four groups **(A)** and, respectively, in DLG **(B)**, DLS **(C)**, TRG **(D)**, and TRS **(E)**. Three samples in each group, each example = 10 fish. The hierarchical clustering tree was calculated using the UPGMA (Unweighted Pair-group Method with Arithmetic Mean) method. The relationship between samples was determined by Bray distance and the average clustering method.

The main strains in the four groups (DLG, DLS, TRG, and TRS) are shown in Figure 4A. According to the relative abundance of strains, four groups are arranged as follows from rich to poor: (a) For DLG (Figure 4B), *Planctomycetes* (19.68%) > *Actinobacteria* (17.78%) > *Oxyphotobacteria* (17.47%) > *Proteobacteria* (16.85%) > *Firmicutes* (10.57%) > *Verrucomicrobia* (9.89%) > *Bacteroidetes* (3.68%) > others (2.53%) > Deinococcus-Thermus (0.92%) > *Tenericutes* (0.62%) > *Armatimonadetes* (0.01%); (b) for DLS (Figure 4C) accounting for more than 10%, *Actinobacteria* (28.23%) > *Bacteroidetes* (25.58%) > *Oxyphotobacteria* (21.40%) > *Proteobacteria* (16.39%); (c) for TRG (Figure 4D), *proteobacteria* occupied an absolute advantage of 63.89%, *Firmicutes* account for 20.50% DLG, and the total proportion of other bacteria is 15.61%; (d) for TRS (Figure 4E), the proportion of main fungi was relatively average, and four kinds of bacteria accounting for more than 20% were dominant: *Oxyphotobacteria* (24.98%) > *Proteobacteria* (23.15%) > *Actinobacteria* (22.14%) > *Bacteroidetes* (20.85%); and (e) the clustering results of the four groups of samples revealed little difference between each group, (Figures 4F,G), indicating that the detection results are highly correlated and reliable.

### Screening of Sensitive Microorganisms to Determine Survival Status

**Figure 5:**
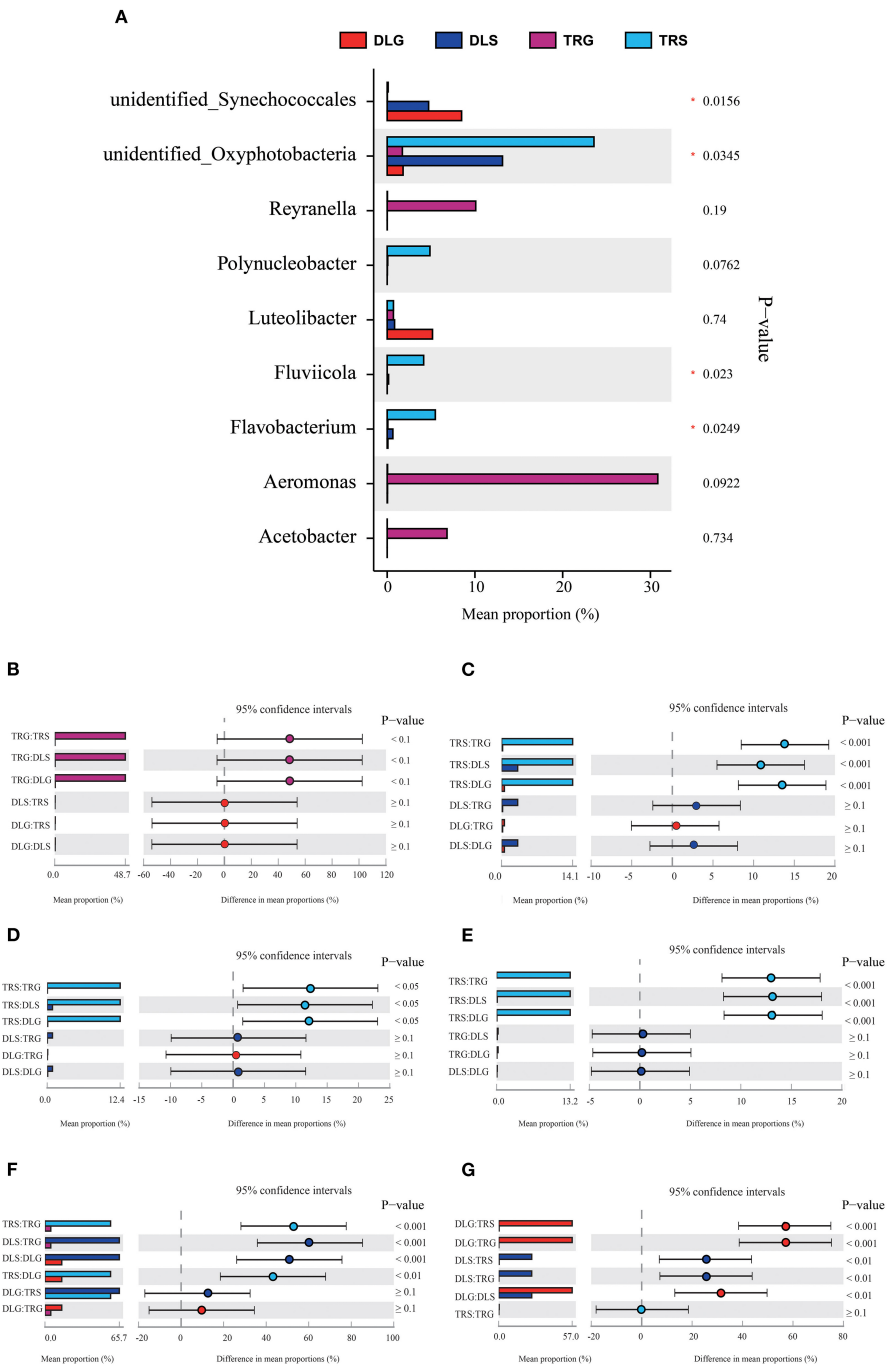
Comparison of bacterial abundances in intestine and water at the alkaline water and freshwater environment at the genus level **(A)**. One-way ANOVA bar plot on genus level for *Aeromonas* **(B)**, *Flavobacterium* **(C)**, *Fluviicola* **(D)**, *Polynucleobacter* **(E)**, *unidentified_Synechococcales* **(F)**, and *unidentified_ Oxyphotobacteria* **(G)**. * stands for 0.01 < *P* ≤ 0.05.

**Figure 6:**
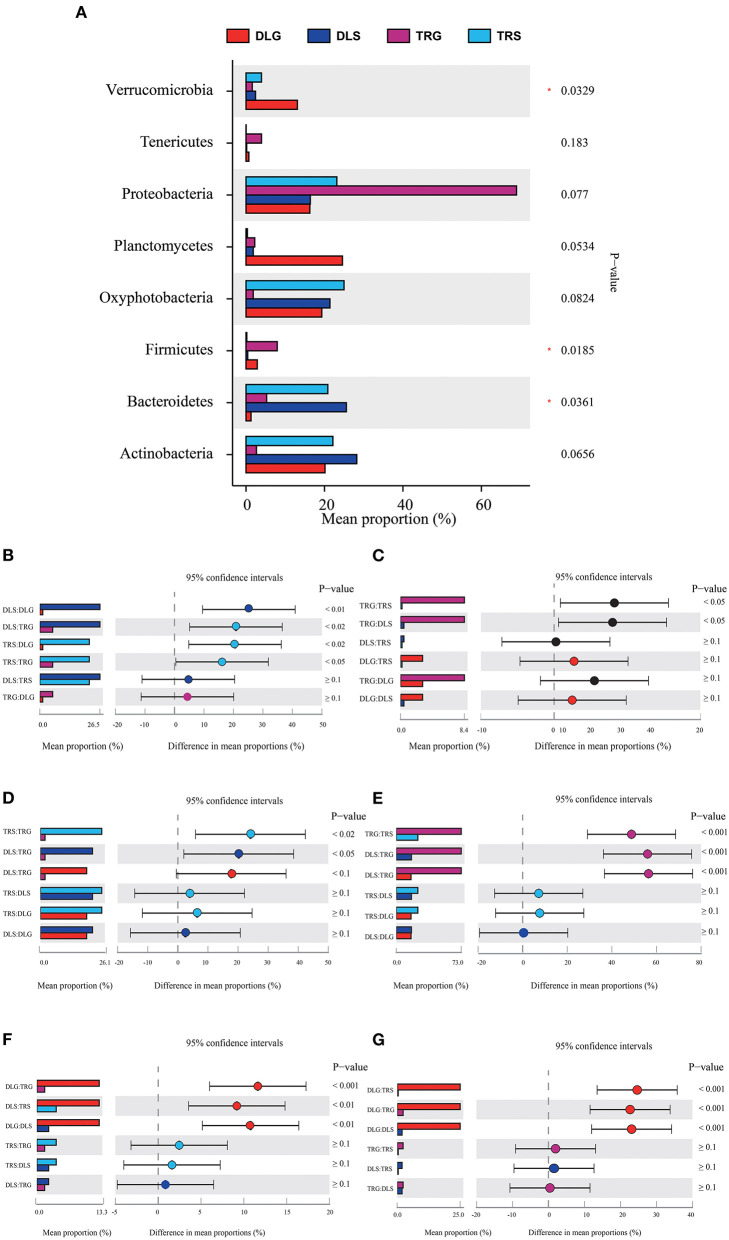
Comparison of bacterial abundances in intestine and water at the alkaline water and freshwater environment at the phylum level **(A)**. One-way ANOVA bar plot on phylum level for *Bacteroidetes* **(B)**, *Firmicutes* **(C)**, *Oxyphotobacteria* **(D)**, *Proteobacteria* **(E)**, *Verrucomicrobia* **(F)**, and *Planctomycetes* **(G)**. *stands for 0.01 < *P* ≤ 0.05.

Several researchers have applied a multinomial logit model to analyze the forecasting accuracy of indicator populations and discovered that they would have highly accurate results at the *bacteroideae* level (Xiong et al., 2014). Following this method, the indicative evaluation in this study was used to filter the variations in the bacterial genus (Figure 5) and phylum (Figure 6), reflecting the survival status of AW and FW in alkaline water and freshwater, respectively. We chose communities with indicator values > 0.90. We screened 15 microbial families to increase the accuracy of the results, displaying the characteristics of indicator families in all samples. At the genus level, four genera of nine dominant genera, summarized in Figure 5A, showed statistically significant differences (*P* < 0.05) in all samples, namely, *Aeromonas* (Figure 5B), *Flavobacterium* (Figure 5C), *Fluviicola* (Figure 5D), *Unidentified_Synechoccales* (Figure 5F), and *Unidentified_Oxyphotobacteria* (Figure 5G). There were significant differences (*P* < 0.001) in TRS, DLS, and DLG of the *Flavobacterium* and *Polynucleobacter* compared with TRG, but there were no statistically significant differences between the three groups of DLS:TRS, DLG:TRS, and DLG:DLS. In *Fluviicola*, the change was similar to the former. However, the significant increase was lower (*P* < 0.05) in the TRS:TRG, TRS:DLG, and TRS:DLG groups. In addition, three groups (TRS:TRG, DLS:TRG, and DLS:DLG) in the *Unidentified_Synechoccales* showed significant differences (*P* < 0.001), and TRS:DLG showed a substantial increase (*P* < 0.01) in the *Unidentified_Oxyphotobacteria*, additional information about the relative abundances at genus level among the four groups can be acquired in Appendix S2.

Three of the eight dominant phyla showed statistically significant differences (*P* < 0.05) in all samples (Figure 6A), namely, *Bacteroidetes* (Figure 6B), *Firmicutes* (Figure 6C), and *Verrucomicrobia* (Figure 6F). There were significant differences (*P* < 0.001) in TRS, DLS, and DLG of the *Proteobacteria* (Figure 6E) compared with TRG, and slight trends of TRS (*P* < 0.05), DLS (*P* < 0.05), and DLG (*P* < 0.1) in *Oxyphotobacteria* (Figure 6D) compared with TRG. However, there were no statistically significant differences between the three groups (DLS:TRS, DLG:TRS, and DLG:DLS). In *Planctomycetes* (Figure 6G), the change was similar to that of the former. However, a significant increase was observed (*P* < 0.001) in the three groups DLG:DLS, TRS:DLG, and TRS:DLG. Four groups (DLS:DLG, DLS:TRG, TRS:DLG, and TRS:TRG) in the *Bacteroidetes* showed significant differences (*P* < 0.05), and two groups (TRG:TRS and TRG:DLS) increased at a similar level in the *Firmicutes*, and groups of TRG:TRS and TRG:DLS showed a significant increase (*P* < 0.05). In *Verrucomicrobia*, there were significant increases in DLG:TRG (*P* < 0.001), DLS:TRS (*P* < 0.05), and DLG:DLS (*P* < 0.05), additional information about the relative abundances at phylum level among the four groups can be acquired in Appendix S3.

### Functional Prediction

**Figure 7:**
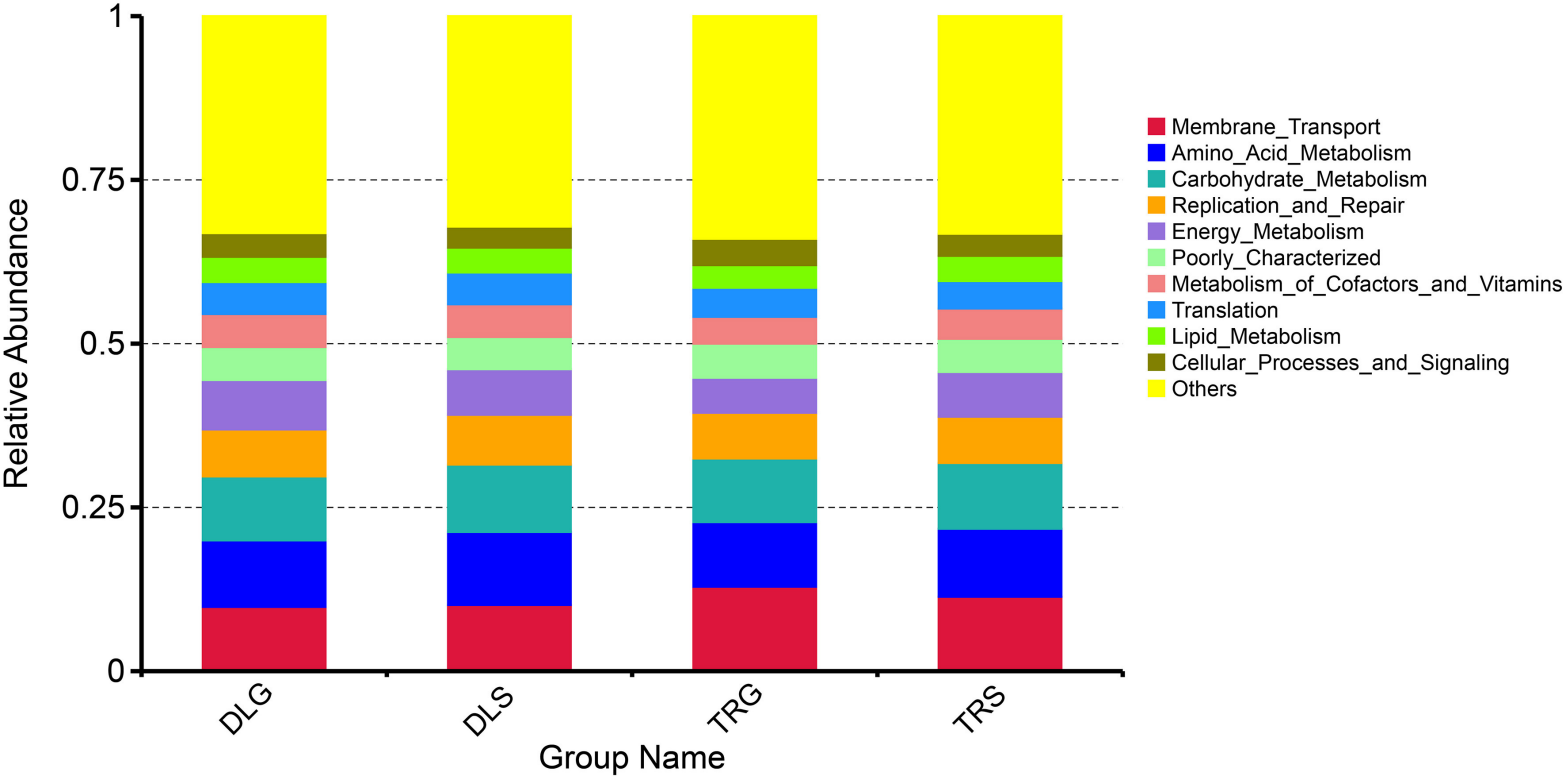
The KEGG function prediction of the four groups.

PICRUSt illustrated significantly changed functions of intestinal microbiota in shrimp intestines and living waters. Compared with the taxonomic profiles, the main functions checked from the samples were broken into 11 parts that showed more similarity in all four groups. The projected capabilities can be prioritized to define the function of gut microbes based on the percentage summarized in Figure 7: unknown function (33.12–33.99%) > membrane transport (9.81–12.89%) > amino acid metabolism (9.87–11.15%) > carbohydrate metabolism (9.71–10.28%) > replication and repair (6.98–7.61%) > energy metabolism (5.35–7.55%) > poorly characterized (4.92–5.20%) > metabolism of cofactors and vitamins (4.10–5.04%) > translation (4.19–4.88%) > lipid metabolism (3.45–3.86%) > cellular processes and signaling (3.21–4.02%). These results showed that the gastrointestinal microbiome of shrimp exhibits biological roles similar to those found in alkaline water or freshwater. Many metabolic activities are aided by microbiome-like membrane transport, amino acid metabolism, glucose metabolism, energy metabolism, replication, and repair.

## Discussion

High-throughput screening technology is widely used to detect a few microbial populations. Compared to previous studies that detected gut microbes, such as traditional culture techniques and bacterial 16S rRNA gene-based DGGE and T-RFLP, such screening can accurately respond to microbial community composition (Ingham et al., 2007; Harrison et al., 2010). In this study, we used Illumina, a high-throughput sequencing technology, to examine intestinal bacterial community composition differences between AW and FW of *L. waleckii* in their living environments (alkaline water and freshwater, respectively).

Some studies have shown that intestinal microorganisms have a huge effect on the host's development and growth, which can not only maintain appropriate energy yield from the diet but can also imply biodiversity and provide constructive forecasting to the continuously shifting intestinal microecological circumstances (Yano et al., 1995; Turnbaugh et al., 2006; Tremaroli and Bäckhed, 2012). The intestinal bacterial variety of *L. waleckii* from alkaline water and freshwater ecosystems was investigated in this study, finding that *Moraxella osloensis, Psychrobacter maritimus*, and *Psychrobacter faecalis* were the most abundant phylum of *L. waleckii* gut bacteria in alkaline water. *Pseudomonas anguilliseptica, Nitrincola nitratireducens, Rhodonellum ikkaensis, Flavobacterium lacus*, and *Alishewanella tabrizica* were significantly enriched in the alkaline water ecosystem. Among them, *Psychrobacter sp*. in the gastrointestinal tract of groupers can be used as a probiotic by inhibiting the growth of a variety of common pathogens. Adding this bacterial species to the feed can significantly improve the food utilization rate of groupers and enhance the immune function of animals (Sun et al., 2011, 2014; Zhang et al., 2020). *Psychrobacter maritimus, P. faecalis, and P. anguilliseptica* belonged to psychrophilic *Bacillus* in this study, meaning that they are the dominant bacteria in the intestinal contents of fish. *Psychrophilic Bacillus* belongs to hypothermic bacteria and has vigorous growth activity even in a refrigerated environment, which has great application value for developing hypothermic microorganisms. The results were verified in *Brachymystax lenok* (Huang et al., 2018). In this study, there was no significant difference in the relative abundance of most phylum in *L. waleckii* gut bacterial communities in alkaline water and freshwater.

Aquatic organisms, growth, ecosystem surroundings, feed and feeding strategies, and digestive physiological properties influence the composition, quantity, and structure of the fish gut microbiome. Although the design and proportions of the intestinal microbiota group in the early stages of fish advancement are still unknown (Bakke et al., 2015), it is speculated that the obliteration of the dynamic equilibrium of gut bacteria is frequently accompanied by the emergence of host diseases (Allison and Martiny, 2008; Werner et al., 2011). Although the intestinal flora of different fish species differs, these microbial communities have similar bioactivities (Sullam et al., 2012; Li et al., 2015). This study discovered that adjustments in gut bacteria were preceded by substantial changes in the intestinal flora in AW and its alkaline hydrosphere. However, there was little difference in the microbial mixtures of different alkaline water and freshwater environments. This demonstrates a significant positive correlation between the structural and functional similarity of bacterial communities and that the functional redundancy of *L. waleckii* intestinal flora is relatively low. As a result, the predicted practical genomic change was consistent with the morphology of the microbial colony in the aquatic environment. In this study, the functions of intestinal flora in AW of Dali Nor-Lake were primarily focused on transcription, methane metabolism, and amino acid metabolism.

Meanwhile, the parts of the intestinal flora in FW of the Tangwang River mainly focus on ion transport, protein repair, and bacterial flow. This indicates that the abundance of bacteria is reduced throughout AW in the physiological process of high alkalinity resistance, accompanied by a weakened ability of multiple energy metabolism. In addition, physiological and metabolic functions are slowed. Thus, AW is involved in many digestive and physiological absorption processes, such as amino acid and carbohydrate metabolism, to provide adequate time to adapt to the highly alkaline water gradually.

Furthermore, most microbial functions in the highly alkaline water of Dari Nor-Lake were focused on amino acid metabolisms, such as serine, glycine, and threonine. In contrast, FW microbial parts in the freshwater of the Tangwang River were primarily focused on pyruvate and cholesteryl butyrate metabolism, which indicates that AW promotes amino acid metabolism and indirectly accelerates enzyme activity in the process of alkalinity tolerance in highly alkaline water. For example, peroxidase can use the redox reaction of catalase to play a detoxification role, and catalase is also involved in lipid synthesis (Lincoff et al., 2007; Wilcox et al., 2008). Thus, the emergence methodology of a high alkalinity tolerance may be associated with changes in the intestinal flora, which relieves digestive absorption and energy metabolism-related functions so that AW generates a sharp sense of the highly alkaline water, allowing enough time for adaptation. We cannot currently establish the causality of the gut community composition, function, and increased alkalinity tolerance due to a lack of references, but their close correlation demonstrated here confirms the critical role of gut microbes in maintaining high alkalinity tolerance in the host, which warrants further investigation.

## Conclusion

There are significant differences in the intestinal microbial community structure and the massive metabolic changes between AW of *L. waleckii* living in an alkaline environment and FW of *L. waleckii* living in a freshwater habitat, indicating that the intestinal microorganisms have low functional redundancy. These changes are consistent with their growth waters. Simultaneously, sensitive intestinal indicator bacteria were tested for survival. Although we cannot currently establish a causal relationship between intestinal community composition, function, and living waters, the close relationship between these three parameters confirms the critical role of digestive microorganisms in host physiology.

## Data Availability Statement

The datasets presented in this study can be found in online repositories. The names of the repository/repositories and accession number(s) can be found below: https://www.ncbi.nlm.nih.gov/bioproject/PRJNA810745.

## Ethics Statement

The animal study was reviewed and approved by the Animal Care and Use Committee of Heilongjiang River Fisheries Research Institute of Chinese Academy of Fishery Sciences (HRFRI).

## Author Contributions

LLi and YC conceptualized the study. LLi and YX performed experiments and data analysis. LLu and YX wrote the manuscript, with suggestions from BS, LZ, and ZZ. All the authors approved the manuscript.

## Funding

This study was supported by the Central-Level Non-Profit Scientific Research Institutes Special Funds (HSY202005M), the National Key Research and Development Project (2020YFD0900402, 2019YFD0900405), the basic scientific research business expenses project of Chinese Academy of Fishery Sciences (2020TD22), and the China Scholarship Council in 2021 (202108310020).

## Conflict of Interest

The authors declare that the research was conducted in the absence of any commercial or financial relationships that could be construed as a potential conflict of interest. The handling editor declared a shared affiliation with the authors LLu, YC, BS, LZ, ZZ, and LLi at the time of the review.

## Publisher's Note

All claims expressed in this article are solely those of the authors and do not necessarily represent those of their affiliated organizations, or those of the publisher, the editors and the reviewers. Any product that may be evaluated in this article, or claim that may be made by its manufacturer, is not guaranteed or endorsed by the publisher.
