# Erratum: Microbiota comparison of Amur ide (*Leuciscus waleckii*) intestine and waters at alkaline water and freshwater as the living environment

**DOI:** 10.3389/fmicb.2022.1027214

**Published:** 2022-09-09

**Authors:** Frontiers Production Office

**Affiliations:** Frontiers Media SA, Lausanne, Switzerland

**Keywords:** *Leuciscus waleckii*, intestine, microbiota, alkaline water, freshwater

Due to an editorial error, the article was published in the incorrect journal section. This article's specialty section has been corrected to Microbial Symbioses, a section of the journal *Frontiers in Microbiology*.

The publisher apologizes for this mistake. The original article has been updated.

