# Supplementary material for: Microbiota Comparison of Amur ide (Leuciscus waleckii) Intestine and Waters at Alkaline Water and Freshwater as the Living Environment
Source: Front Microbiol. 2022 May 4;13:881132. doi: 10.3389/fmicb.2022.881132 (PMC9114670; doi:10.3389/fmicb.2022.881132)
Supplement: Supplementary file 1 [file Data_Sheet_1.ZIP › Supplementary data/Supplementary Table 1.docx]

**TABLE S1** Raw sequences information

| **ID** | **Raw reads** | **Base (nt)** | **Average length (nt)** | **Q20** | **GC%** | **Effective (%)** |
| --- | --- | --- | --- | --- | --- | --- |
| DLG1 | 54806 | 13087590 | 252 | 84.17 | 54.24 | 94.69 |
| DLG2 | 85261 | 20301193 | 252 | 89.98 | 54.41 | 94.2 |
| DLG3 | 90490 | 20938513 | 252 | 85.11 | 54.65 | 91.7 |
| TRG1 | 87636 | 20295188 | 253 | 87.7 | 52.71 | 91.49 |
| TRG2 | 57407 | 13885442 | 252 | 81.17 | 51.66 | 95.71 |
| TRG3 | 84448 | 20266787 | 252 | 87.11 | 53.35 | 94.95 |
| DLS1 | 86289 | 20250983 | 252 | 89.89 | 53.39 | 92.88 |
| DLS2 | 62821 | 14344340 | 251 | 84.85 | 52.72 | 90.67 |
| DLS3 | 87380 | 20305568 | 252 | 90 | 53.43 | 92.01 |
| TRS1 | 86634 | 20245136 | 252 | 84.84 | 50.37 | 92.6 |
| TRS2 | 83209 | 20321271 | 253 | 76.73 | 52.65 | 96.37 |
| TRS3 | 81762 | 18834370 | 252 | 84.4 | 50.13 | 91.24 |
